# Identification of risk factors for acute exacerbation of idiopathic pulmonary fibrosis based on baseline high-resolution computed tomography: a prospective observational study

**DOI:** 10.1186/s12890-024-03172-w

**Published:** 2024-07-19

**Authors:** Zhaojun Wang, Zhengping Zhang, Li Zhu, Jia Hou, Hongyan Fu, Xiaojun Yang, Faxuan Wang, Juan Chen

**Affiliations:** 1 Department of Key Laboratory of Ningxia Stem Cell and Regenerative Medicine, Institute of Medical Sciences, General Hospital of Ningxia Medical University, Yinchuan, Ningxia 750004 China; 2 Department of Pulmonary and Critical Care Medicine, General Hospital of Ningxia Medical University, Yinchuan, Ningxia 750004 China; 3 Department of Critical Care Medicine, General Hospital of Ningxia Medical University, Yinchuan, China; 4 Department of Radiology, General Hospital of Ningxia Medical University, Yinchuan, China; 5 School of Public Health and Management, Ningxia Medical University, Yinchuan, China

**Keywords:** Acute exacerbation of idiopathic pulmonary fibrosis, CT-derived quantitative parameters, Risk factors, Prospective observational study

## Abstract

**Background:**

This study aimed to investigate risk factors for acute exacerbation of idiopathic pulmonary fibrosis (AE-IPF) based on baseline high-resolution computed tomography (HRCT).

**Methods:**

This prospective observational study enrolled patients with IPF treated at the General Hospital of Ningxia Medical University between January 2019 and January 2021. HRCT-derived quantitative parameters at baseline were analyzed.

**Results:**

A total of 102 patients [92 (90.2%) males with a mean age of 67 years] with IPF were included, with a median follow-up of 32 (24-40.5) months. AE occurred in 30 (29.4%) IPF patients. Multivariable logistic regression analysis identified Doppler transthoracic echocardiography suggestive of pulmonary hypertension (PH) (13.43; 95% CI: 4.18–41.09; *P* < 0.001), honeycombing (OR 1.08; 95% CI: 1.02–1.14; *P* = 0.013), and whole lung volume (OR 0.99; 95% CI: 0.99-1.00; *P* = 0.037) as independent risk factors for AE-IPF. The combination of PH, honeycombing, whole lung volume, and the percentage of predicted forced vital capacity (FVC% pred) showed a high area under the curve from receiver operating characteristic curves of 0.888, with a sensitivity of 90% and specificity of 78%.

**Conclusions:**

This study emphasizes that quantitative CT parameters (honeycombing, whole lung volume) may serve as risk factors for AE-IPF. The combination of honeycombing, whole lung volume, FVC% pred, and PH may aid in predicting AE-IPF.

**Supplementary Information:**

The online version contains supplementary material available at 10.1186/s12890-024-03172-w.

## Background

Idiopathic pulmonary fibrosis (IPF), accounting for 17-86% of all interstitial lung diseases (ILD) cases, is a chronically progressive fibrosing interstitial pneumonia of unknown etiology, which associates with histopathological and/or radiological pattern of usual interstitial pneumonia (UIP) [1]. This condition, characterized by gradual worsening of dyspnea and irreversible loss of pulmonary function, primarily affects the aged, with a median survival of 3–5 years after diagnosis [2–4]. Notably, the natural course of IPF is eminently heterogeneous and unexpected, with the majority cases advancing slowly over time and a minority deteriorating rapidly and even dying in few months. Furthermore, a group of IPF patients may suffer intermittent acute respiratory aggravation for idiopathic factor or triggers–termed “acute exacerbations of IPF (AE-IPF), with an in-hospital mortality rate exceeding 50% [5–8] and being responsible for over 46% IPF mortality [9]. Predicting disease progression of IPF is important for prevention and therapeutic management.

The pathogenesis of AE in IPF patients remains elusive and the onset of AE-IPF is highly unpredictable, featuring new bilateral ground-glass opacities (GGO) and/or consolidations at high-resolution computed tomography (HRCT) against a background pattern in line with fibrosing ILD [10]. Currently, some risk factors for the occurrence of AE-IPF have been identified [11–15], including pulmonary function parameters, biomarkers, and radiological signs [16]. Given its accessibility and widespread availability, HRCT of the chest holds significant promise as a non-invasive method for assessing the condition and predicting the prognosis of patients with IPF. A study revealed that the baseline extent of fibrosis and GGO on HRCT images were associated with AE-IPF [16]. Furthermore, the development of quantitative CT makes it more precise to predict AE onset for patients with IPF, especially with utilization of automated quantification. Nam and colleagues showed that CT-quantified volumetric parameters, such as normal lung volume and extent of fibrosis, were correlated with physiologic variables and can be served as independent predictors for overall survival of IPF patients [17]. However, the characteristic of CT-derived quantitative parameter and its role in the assessment of AE-IPF patients are still not clearly elucidated, and prospective investigations are in demand to confirm such relationship.

This study aimed to investigate risk factors for AE-IPF based on baseline HRCT.

## Methods

### Study design and patients

This prospective observational study was conducted at the General Hospital of Ningxia Medical University between January 2019 and January 2021, enrolling patients diagnosed with IPF. Inclusion criteria were as follows: (1) Patients diagnosed with IPF according to the American Thoracic Society (ATS)/European Respiratory Society (ERS) consensus guidelines [3]; (2) Patients aged 18–75 years; (3) Patients able to cooperate with medical staff to complete necessary assessments and evaluations; (4) Patients with a time interval of less than three months between PFT and HRCT; (5) Voluntary participation and signed informed consent. Exclusion criteria were: (1) Patients with chronic obstructive pulmonary disease, bronchial asthma, sleep apnea hypoventilation syndrome, bronchiectasis, tuberculosis, and other lung diseases causing chronic airway obstruction; (2) Patients with interstitial lung diseases devoid of fibrotic processes, such as amyloidosis, occlusive bronchiectasis with opportunistic pneumonia, ferritins, alveolar proteinosis, and lymphangioleiomyomatosis; (3) Critically ill patients with multi-organ failure involving the heart, liver, kidney, and other organs; (4) Patients with acute coronary syndrome and acute cerebrovascular disease; (5) Patients with mental disorders. This study adhered to ethical protocols approved by the Ethics Committee for the Conduct of Human Research (No. KYLL-2019-455). All participants were informed about potential study risks and provided written informed consent.

### Procedure and follow-up

Volumetric HRCT scans of the chest were conducted at enrollment, the 6-month follow-up, the 1-year follow-up, upon AE occurrence, and during the review after AE treatment. These scans were obtained using Lightspeed Ultra (GE Healthcare, Chicago, IL), Somatom Sensation 16 (Siemens Healthineers, Erlangen, Germany), and Philips Brilliance 16 (Philips Medical Systems, Best, Netherlands). Images covering the entire lung from apex to base were acquired in the supine position during full inspiration.

The scanning protocol involved a standard-dose CT with a slice thickness of less than 1 mm. CT scan parameters included peak voltage of 120 kVp, tube current modulation ranging from 100 to 200 mAs, and Bone kernel for GE, B50-70f for Siemens, and YA kernel for Philips. Prior to the HRCT examination, an experienced radiologist provided training and instructions to patients, ensuring correct execution of all breathing maneuvers.

Quantitative assessment of HRCT was performed using commercial deep learning software (AVIEW, Coreline Soft). The software automatically classified parenchymal patterns, including emphysema, consolidation, honeycombing, reticulation, GGO, and normal lung (Supplementary  1). Parenchymal patterns were expressed as proportions (%), whole lung volume was measured in cubic centimeters (CC).

Under the supervision of a certified pulmonary technologist, PFTs were conducted in accordance with the guidelines of the American Thoracic Society and European Respiratory Society (ERS) [18]. These tests were performed at baseline, with data collected on the percentage of predicted forced vital capacity (FVC% pred) and the percentage of predicted diffusing capacity for carbon monoxide (DLCO% pred).

Baseline demographics and clinical characteristics, encompassing age, sex, body mass index (BMI), smoking status, gender, age, GAP score, lung function, partial pressure of oxygen (PaO_2_) / fraction of inspiration oxygen (FiO_2_), laboratory results, surgical lung biopsy, HRCT images, pharmacologic treatments, and comorbidities (pulmonary hypertension, diabetes, coronary heart disease, emphysema, lung cancer), were extracted from hospital medical records.

The disease course of IPF was defined as the duration from the onset of cough or shortness of breath symptoms to the initial visit for IPF diagnosis. Regular follow-up was conducted via telephone or WeChat every three months. The follow-up period commenced from the initial IPF diagnosis and continued until the study’s completion in June 2023. The diagnosis of AE-IPF adhered to criteria established by the International Working Group (2016). The updated guidelines introduced the concepts of both triggered and idiopathic AE. Triggered AE referred to cases where AE onset was prompted by specific events or factors, such as infection or other underlying triggers (e.g., aspiration, drug toxicity, postoperative) [6].

Doppler transthoracic echocardiography was performed at enrollment, with cardiac ultrasound conducted annually thereafter. Doppler transthoracic echocardiography suggestive of pulmonary hypertension (PH) was defined as either a peak tricuspid velocity of ≥ 3.4 m/s or a peak tricuspid velocity of ≥ 2.9 m/s accompanied by at least two out of three echocardiographic indicators of PH, in accordance with the 2015 ESC/ERS guidelines [19].

### Statistical analysis

Continuous variables were presented as mean ± standard deviation (SD) or median (interquartile range (IQR)), while categorical variables were described as numbers (percentage). Correlations between variables were analyzed using Pearson’s or Spearman’s test. Univariable analysis was conducted to explore associations between variables and AE-IPF, with variables showing *P* < 0.1 included in multivariable logistic regression analysis. In the multivariate analysis, highly correlated variables with Spearman’s or Pearson’s correlation coefficients > 0.6 were excluded to avoid multicollinearity. Factors were entered into the multivariate logistics regression model using backward stepwise selection with the Akaike information criterion (AIC). Comparative analysis of predictive models for AE-IPF utilized combined risk factors and was assessed using the Delong test. Model 1 included PH, honeycombing, and whole lung volume; Model 2 included PH, honeycombing, whole lung volume, and FVC% pred; Model 3 included PH, honeycombing, whole lung volume, and DLCO% pred; Model 4 included PH, honeycombing, whole lung volume, DLCO% pred, and FVC% pred; Model 5 included PH, honeycombing, DLCO% pred, and FVC% pred.

The discrimination of predictive models for AE-IPF onset was assessed using the area under the curve (AUC) derived from receiver operating characteristic (ROC) curves. Optimal cut-off values were identified through the Youden index. Calibration and decision curve analyses were conducted to evaluate the model’s calibration performance and clinical utility. The model’s calibration was further evaluated with the Hosmer-Lemeshow test. Internal validation was achieved through bootstrapping with 500 resamples. All data were analyzed using SPSS version 26.0 (IBM, Armonk, NY), and R software (Version 4.3.2). Two-sided p-values < 0.05 were considered statistically significant.

## Results

### Baseline characteristics

**Fig. 1:**
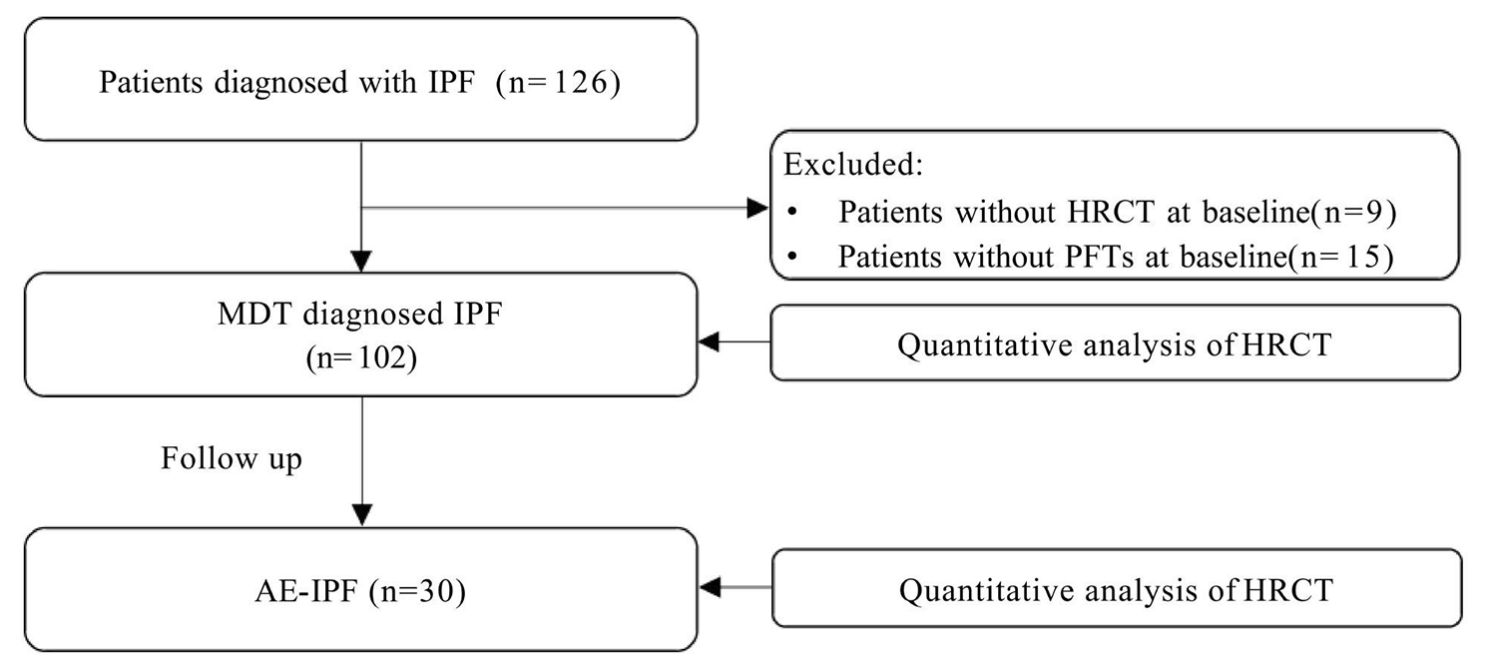
Study flow chart. AE, acute exacerbation, HRCT, high-resolution computed tomography; IPF, idiopathic pulmonary fibrosis; MDT, multidisciplinary teams

Initially, 126 IPF patients were enrolled, with nine participants excluded due to a lack of high-quality chest HRCT images and 15 excluded for non-IPF diagnoses. Thus, a total of 102 patients [92 (90.2%) males, mean age 67 years] with IPF were included. The median disease duration of IPF was 24 months (IQR: 12–48), and the median follow-up time was 32 months (IQR: 24-40.5). Throughout the follow-up period, AE occurred in 30 of 102 IPF patients (29.4%), with 20 of 30 (66.7%) classified as triggered AE and the remaining 10 of 30 (33.3%) as idiopathic AE (Fig. 1).

Compared to the non-AE-IPF group, patients with AE-IPF were more likely to have pulmonary hypertension [20 (66.7%) vs. 9 (12.5%), *P* < 0.001] and had significantly lower FVC% pred (76.5 ± 18.3 vs. 85.8 ± 16.0, *P* = 0.011) and DLCO% pred (46.7 ± 17.8 vs. 57.2 ± 20.1, *P* = 0.015). Regarding CT-derived quantitative parameters, patients in the AE-IPF group had significantly lower normal lung percentages [60.0 (42.6–76.4) vs. 74.2 (61.8–86.4), *P* < 0.001] and whole lung volumes (3364 ± 748 vs. 4020 ± 1068, *P* < 0.001), and more consolidation [0.06 (0.01–0.26) vs. 0.02 (0.01–0.08), *P* = 0.027], reticulation [16.4 (10.9–25.9) vs. 9.94 (4.17–18.5), *P* = 0.004], and honeycombing [8.2 (2.2–16.4) vs. 1.5 (0.1–7.4), *P* < 0.001] compared to patients in the non-AE-IPF group. There were no significant differences between the two groups in terms of demographics, oxygenation, antifibrotic therapy, GAP scores, and radiological signs of emphysema and GGO (all *P* > 0.05) (Table 1).

**Table 1:** General characteristics and CT-derived quantitative parameters at baseline

Variables	Total IPF (*N* = 102)	AE-IPF (*N* = 30)	Non-AE-IPF (*N* = 72)	*P*
Age (years)	67 ± 8	69 ± 9	67 ± 8	0.366
Male: female (*n*)	92:10	28:2	64:8	0.719
BMI (kg/m^2^)	24.5 ± 3.3	23.7 ± 3.6	24.9 ± 3.2	0.089
Smoking status, *n* (%)				
Never smoking	31 (30.4%)	7 (23.3%)	24 (33.3%)	0.355
Smoking or ex-smoking	71 (69.6%)	23 (76.7%)	48 (66.7%)	
PaO_2_/FiO_2_(mmHg)	309.7 ± 78.4	304.6 ± 96.8	311.9 ± 69.8	0.669
Surgical Lung biopsy, *n* (%)	3 (2.9%)	2 (6.7%)	1 (1.4%)	0.206
Antifibrotic therapy, *n* (%)	95 (93.1%)	29 (96.7%)	66 (91.7%)	0.670
GAP score	3 (3–4)	4 (3–5)	3 (3–4)	0.057
Disease course (months)	24.0 (12.0–48.0)	16.5 (11.8–39.0)	35.5 (12.0–48.0)	0.530
Follow-up duration (months)	32.0 (24.0-40.5)	32.0 (24.0-40.5)	32.0 (24.0-42.5)	0.604
**Comorbidities**				
Pulmonary hypertension	29 (28.4%)	20 (66.7%)	9 (12.5%)	< 0.001
Diabetes	13 (12.7%)	5 (16.7%)	8 (11.1%)	0.518
Coronary heart disease	16 (15.7%)	6 (20.0%)	10 (13.9%)	0.551
Emphysema	73 (71.6%)	22 (73.3%)	51 (70.8%)	0.989
Lung cancer	10 (9.80%)	5 (16.7%)	5 (6.94%)	0.154
**Pulmonary function tests**				
FVC % predicted	83.1 ± 17.2	76.5 ± 18.3	85.8 ± 16.0	0.011
DLCO % predicted	54.1 ± 19.9	46.7 ± 17.8	57.2 ± 20.1	0.015
**CT-derived quantitative parameters**				
Normal lung (%)	71.4 (54.5–82.6)	60.0 (42.6–76.4)	74.2 (61.8–86.4)	0.001
Emphysema (%)	0.7 (0.2–2.2)	0.9 (0.4–3.0)	0.6 (0.1–2.1)	0.233
Consolidation (%)	0.03 (0.01–0.12)	0.06 (0.01–0.26)	0.02 (0.01–0.08)	0.027
GGO (%)	2.28 (0.2–10.2)	2.0 (0.4–7.5)	2.3 (0.2–10.4)	0.892
Reticulation (%)	12.8 (5.24–21.7)	16.4 (10.9–25.9)	9.94 (4.17–18.5)	0.004
Honeycombing (%)	2.6 (0.1–8.8)	8.2 (2.2–16.4)	1.5 (0.1–7.4)	0.001
Whole lung volume (cc)	3827 ± 1026	3364 ± 748	4020 ± 1068	0.001

AE, acute exacerbation; BMI, body mass index; DLCO, diffusing capacity for carbon monoxide; IPF, idiopathic pulmonary fibrosis; FVC, forced vital capacity; GGO, ground-glass opacities; HRCT, high-resolution computed tomography; IPF, idiopathic pulmonary fibrosis

### Correlations between CT-derived parameters and PFTs

Significant correlations were observed between CT-derived parameters and PFTs (Supplementary Fig. 1). Positive correlations were identified between normal lung% and PFTs (DLCO% pred, *r* = 0.49, *P* < 0.05; FVC% pred, *r* = 0.21; *P* < 0.05). Whole lung volume was positively correlated with PFTs (FVC% pred, *r* = 0.42, *P* < 0.05; DLCO% pred, *r* = 0.25, *P* < 0.05). Conversely, negative correlations were observed between reticulation% and PFTs (DLCO% pred, *r* = − 0.37, *P* < 0.05; FVC% pred, *r* = − 0.36, *P* < 0.05).

### Independent risk factors for onset of AE-IPF

In univariable analysis, BMI (OR 0.89; 95% CI: 0.77–1.02; *P* = 0.088), GAP score (OR 1.39; 95% CI: 1.03–1.95; *P* = 0.038), comorbidity of PH (OR 14.00; 95% CI: 5.19–41.38; *P* < 0.001), FVC% pred (OR 0.97; 95% CI: 0.94–0.99; *P* = 0.014), and DLCO% pred (OR 0.97; 95% CI: 0.95-1.00; *P* = 0.018) were associated with AE-IPF. Regarding CT-derived quantitative parameters, normal lung% (OR 0.97; 95% CI: 0.94–0.99; *P* = 0.002), reticulation% (OR 1.05; 95% CI: 1.01–1.09; *P* = 0.011), honeycombing (OR 1.08; 95% CI: 1.03–1.14; *P* = 0.002), and whole lung volume (OR 0.99; 95% CI: 0.99-1.00; *P* = 0.004) were associated with AE-IPF (Table 2).

**Table 2:** Univariable and multivariable analysis for predicting AE-IPF.

Variables	Univariable analysis				Multivariable analysis		
	OR	95% CI	*P*		OR	95% CI	*P*
Age (per 1y)	1.03	0.97–1.08	0.348				
Sex	1.75	0.35–8.77	0.496				
BMI (per 1 kg/m^2^)	0.89	0.78–1.02	0.088		0.95	0.8–1.13	0.554
Smoking status	1.64	0.62–4.37	0.320				
PaO_2_/FiO_2_ (per 1mmhg)	1.00	0.99–1.00	0.666				
Surgical lung biopsy	5.071	0.442–58.186	0.192				
Antifibrotic	2.636	0.304–22.897	0.379				
GAP score (per 1)	1.399	1.018–1.923	0.038		0.896	0.52–1.55	0.693
FVC% pred (per 1%)	0.97	0.94–0.99	0.014		0.972	0.938–1.008	0.124
DLCO% pred (per 1%)	0.97	0.95–1.00	0.018		0.994	0.96–1.03	0.716
Pulmonary hypertension	14.00	4.99–39.27	< 0.001		13.43	4.28–41.9	< 0.001*
Diabetes	1.6	0.48–5.36	0.446				
Coronary heart disease	1.55	0.51–4.73	0.442				
Emphysema	1.13	0.44–2.94	0.799				
Lung cancer	2.68	0.72–10.05	0.144				
Normal lung (per 1%)	0.97	0.94–0.99	0.002				
Emphysema (per 1%)	1.003	0.951–1.057	0.923				
Consolidation (per 1%)	1.064	0.522–2.168	0.865				
GGO (per 1%)	1.01	0.97–1.04	0.783				
Reticulation (per 1%)	1.05	1.01–1.09	0.011		0.996	0.93–1.07	0.918
Honeycombing (per 1%)	1.08	1.03–1.14	0.002		1.081	1.02–1.143	0.013*
Whole lung volume (per 1 cc)	0.99	0.999–1.00	0.004		0.999	0.999-1	0.037*

Abbreviation: AE, acute exacerbation; BMI, body mass index; CI, confidence interval; DLCO, diffusing capacity for carbon monoxide; FVC, forced vital capacity; GGO, ground-glass opacities; IPF, idiopathic pulmonary fibrosis; OR, odds ratio. *: *P* ≤ 0.05

In multivariable analysis, comorbidity of PH (OR 13.43; 95% CI: 4.18–41.09; *P* < 0.001), honeycombing (OR 1.08; 95% CI: 1.02–1.14; *P* = 0.013), and whole lung volume (OR 0.99; 95% CI: 0.99-1.00; *P* = 0.037) were associated with AE-IPF (Table 2). Optimal cut-off values for honeycombing and whole lung volume were determined by ROC analysis. The AUC of ROC curves for honeycombing and whole lung volume in predicting AE-IPF were 0.701 (95%CI: 0.586–0.802) and 0.696 (95%CI: 0.591–0.802), respectively. The optimal cut-off value of honeycombing was 7.70 with a sensitivity of 60% and specificity of 78%; the optimal cut-off value of whole lung volume was 3851 cc, with a sensitivity and specificity of 62.5% and 80%, respectively (Supplementary Table 1).

### Models for predicting AE-IPF

Model 1 achieved a ROC-AUC of 0.874, while Model 2 attained a ROC-AUC of 0.888. Model 3 exhibited a ROC-AUC of 0.873, and Model 4 yielded a ROC-AUC of 0.888, Model 5 showed a ROC-AUC of 0.874. Combining PH, honeycombing, whole lung volume, and FVC% pred resulted in a high AUC-ROC of 0.888, with a sensitivity of 90% and specificity of 78%. Comparative analysis did not reveal a statistically significant difference among these models (*P* > 0.05) (Table 3). The Hosmer-Lemeshow test for both models resulted in P values greater than 0.05, indicating no significant discrepancy between the observed and predicted outcomes, as detailed in Table S2. The ROC curve, calibration plot, and Decision curve analysis (DCA) curve were presented in Supplementary Figs. 2, 3, and 4, respectively, to provide a comprehensive visualization of the model’s performance and calibration.

**Table 3:** Comparative analysis of models for predicting AE-IPF utilizing combined risk factors

Models	Sensitivity	Specificity	AUC	Z (vs. model 1)	*P* (vs. model 1)
Model 1	76.7%	88%	0.874	NA	NA
Model 2	90%	78%	0.888	-1.095	0.274
Model 3	76.7%	89%	0.873	0.130	0.897
Model 4	90%	78%	0.888	-1.043	0.297
Model 5	86.7%	83.3%	0.874	-0.021	0.983

Model 1: PH + honeycombing + whole lung volume; Model 2: PH + honeycombing + whole lung volume + FVC% pred; Model 3: PH + honeycombing + whole lung volume + DLCO% pred; Model 4: PH + honeycombing + whole lung volume + FVC% pred + DLCO% pred; Model 5: PH + honeycombing + FVC% pred + DLCO% pred

### Validation of the model

Five models demonstrate equivalent predictive power for AE-IPF, emphasizing the significance of quantitative CT features. We internally validated model 1 using bootstrapping with 500 resamples. Bootstrapped ROC performance: 95% CI:0.792, 0.932 (Supplementary Fig. 5). The calibration plot and DCA curve for model 1 in the internal validation cohort are shown in Supplementary Figs. 6 and 7, respectively.

## Discussion

This study demonstrates that PH, honeycombing, and whole lung volume may independently serve as risk factors for AE-IPF. Additionally, the combination of PH, honeycombing, whole lung volume, and FVC% pred exhibits a high AUC-ROC for predicting AE-IPF. These findings suggest a promising tool for predicting outcomes following AE-IPF.

In recent years, the role of CT parameters in evaluating and predicting prognosis has become increasingly crucial, especially with the development of AI. Accumulating studies have proposed various predictive models for prognosis in IPF patients, often modifying the GAP score. For instance, Chahal et al. incorporated a semiquantitative fibrotic score from thin-section CT into the GAP score, showing improved correlation with transplant-free survival, particularly in patients with GAP score ≤ 3 [20]. However, semiquantitative CT analysis has limitations, such as dependence on radiologists’ expertise and time-consuming analysis. Thus, Wu et al. developed a model predicting mortality with a predictive value exceeding 70%, using percentage of fibrosis and IPF severity determined by FVC% pred, DLCO% pred, SpO2%, age, and gender [21]. Similarly, a retrospective study introduced the GA-FVC-CT index, replacing DLCO% with CT-Norm%, demonstrating comparable discriminative performance in predicting overall survival to the original GAP index [17]. In our study, we also highlight the essential role of CT-derived quantitative parameters in our predictive model, including honeycombing, whole lung volume, FVC% pred, and PH. Notably, our model did not rely on the GAP index, as it was not applicable to severe IPF cases included in our study, characterized by low levels of DLCO% pred and PaO2/FiO2, along with various comorbidities. Furthermore, the inclusion of PH as a generally accepted risk factor in our model enhanced its efficacy in evaluating the onset of AE-IPF. Additionally, Karayama et al. demonstrated a predictive model for AE of ILD, incorporating risk factors such as radiographic honeycombing, age > 75 years, and serum lactate dehydrogenase level > 222 U·L − 1, albeit without utilizing quantitative CT parameters [22].

This study revealed a potential association between BMI and AE-IPF to some degree; however, due to the limited sample size, BMI failed to reach statistical significance in the multivariate analysis. It is well-established that post-hoc analyses of pooled data from the INPULSIS trials suggest that in patients with IPF, lower BMI and weight loss may be linked to a more rapid decline in FVC [23]. Scant research exists on the association between BMI and AE-IPF. The relationship between BMI and AE-IPF warrants further investigation. In a retrospective study, the researchers identified CT-Norm% and CT-Fib% as independent prognostic factors for overall survival in IPF when calculated using chest CT-based deep learning software [17]. Our analysis revealed that CT-derived parameters, including normal lung and reticulation, were associated with an increased risk of AE-IPF in the univariable analysis. However, these parameters did not achieve statistical significance in the multivariate analysis. This discrepancy may be attributed to an insufficient sample size or multicollinearity among the variables.

We believe that the value of quantitative CT in the clinical assessment and management of IPF is reflected in two aspects. Firstly, quantitative parameters of chest HRCT are strongly associated with physiological variables, which are crucial for evaluating diffuse lung diseases, as demonstrated by previous studies [17, 24]. Therefore, quantitative CT could serve as an effective alternative for patients who have difficulty completing PFTs or are unsuitable for this examination. We found a robust positive correlation between normal lung% and DLCO% pred, and a moderate correlation between whole lung volume and FVC % pred. Additionally, we observed significantly negative correlations between reticulation% and physiologic variables. Similarly, a recent study indicated a strong association between DLCO% pred and normal lung% and fibrotic lung% [17].

Secondly, radiological parameters can act as risk factors for IPF. Higher honeycombing and lower whole lung volume were identified as independent predictors of AE onset in IPF patients in this study, suggesting that patients are at high risk for AE-IPF if their CT-derived parameters fall below the cut-off value. Nam G.J. et al. also demonstrated that normal lung% and fibrotic lung% were independent risk factors alongside FVC% pred and DLCO% pred [17]. However, physiological variables were not independent predictive factors in our model. This may be attributed to the strong linear correlation between CT-derived parameters and PFTs in our study, thereby diminishing the role of PFTs. This also suggests the reliability of CT-derived quantitative parameters as alternatives to physiological variables to some extent. Additionally, individuals with a larger extent of fibrosis are likely to experience an earlier onset of AE-IPF [16], and those with a larger honeycombing area may be under more severe conditions, making them more prone to AE requiring hospitalization or unscheduled medical intervention.

Apart from honeycombing and lower whole lung volume, the presence of PH emerges as another independent risk factor for AE-IPF, consistent with prior studies [8, 9, 25–27]. A three-fold increase in mortality has been demonstrated in patients with PH, particularly when systolic pulmonary arterial pressure exceeds 50 mmHg on echocardiography [28]. The development of PH in patients with IPF stems from pulmonary vascular remodeling and vascular smooth muscle cell proliferation, leading to increased pulmonary vascular resistance and afterload on the right ventricle [29]. Consequently, the presence of PH not only compromises survival but also impairs exercise tolerance and quality of life [30]. However, studies have indicated that conventional drugs used in primary pulmonary hypertension (such as bosentan, ambrisentan, macitentan, and riociguat) have yielded unfavorable results in improving hemodynamic abnormalities of PH in patients with IPF. Recent clinical trials have suggested that novel strategies, such as pulmonary vasodilators administered by inhalation and combinations with antifibrotic drugs, may offer a promising approach to addressing this unmet clinical need [31, 32].

This study has several limitations. Firstly, the small sample size of this single-center study, attributed to the low prevalence of IPF and the inability of some IPF patients to undergo HRCT scans throughout the entire AE progression, limited the availability of longitudinal CT-derived data. Therefore, the findings presented in this study require further confirmation through larger multicenter cohorts. Secondly, the utilization of echocardiography instead of right heart catheterization to evaluate PH may lead to misclassification when excluding PH. Thirdly, our study did not employ spirometric gating of CT scans, despite its infrequent use in clinical practice. Fourthly, due to the unprecedented COVID-19 pandemic, a significant proportion of patients failed to adhere to their scheduled outpatient follow-up appointments due to home isolation and restricted social gatherings. To mitigate this challenge, we extended the follow-up period beyond one year. In our upcoming research, we aim to address these limitations to ensure more robust findings. Fifth, our study has yet to undergo external validation. Future research necessitates an expansion of the sample size and the implementation of external validation procedures.

## Conclusions

In conclusion, this study highlights that HRCT parameters may serve as risk factors for AE-IPF. Honeycombing, whole lung volume combined with FVC% pred and PH may be useful for predicting AE-IPF. Multicenter prospective studies with larger samples are needed to validate these results.

### Electronic supplementary material

Below is the link to the electronic supplementary material.

Supplementary Material 1

Supplementary Material 2

Supplementary Material 3

## Data Availability

The datasets used and/or analysed during the current study available from the corresponding author on reasonable request.

## Abbreviations

AE: Acute exacerbation
AUC: Area under the curve
CT: Computed tomography
DLCO: Diffusing capacity for carbon monoxide
ERS: European Respiratory Society
ESC: European Society of Cardiology
FVC: Forced vital capacity
GGO: Ground-glass opacities
HRCT: High-resolution computed tomography
IPF: Idiopathic pulmonary fibrosis
ILD: Interstitial lung disease
PFT: Pulmonary function tests
PH: Pulmonary hypertension
ROC: Receiver operating characteristic
SD: Standard deviation
UIP: Usual interstitial pneumonia
