# Supplementary material for: Identification of risk factors for acute exacerbation of idiopathic pulmonary fibrosis based on baseline high-resolution computed tomography: a prospective observational study
Source: BMC Pulm Med. 2024 Jul 19;24:352. doi: 10.1186/s12890-024-03172-w (PMC11264818; doi:10.1186/s12890-024-03172-w)
Supplement: Supplementary file 1 — Supplementary Material 1 [file 12890_2024_3172_MOESM1_ESM.docx]

**Supplementary table 1** The optimal cut-off values for honeycombing, and whole lung volume.

|  | Cut-off | Sensitivity (%) | Specificity (%) | AUC value (95%CI) |
| --- | --- | --- | --- | --- |
| Honeycombing | 7.70 | 60% | 78% | 0.701(0.586-0.802) |
| Whole lung volume(cc) | 3851 | 62.5% | 80% | 0.696(0.591-0.802) |
| Pulmonary hypertension |  | 66.7% | 88% | 0.771(0.660-0.881) |
