# Supplementary material for: Identification of risk factors for acute exacerbation of idiopathic pulmonary fibrosis based on baseline high-resolution computed tomography: a prospective observational study
Source: BMC Pulm Med. 2024 Jul 19;24:352. doi: 10.1186/s12890-024-03172-w (PMC11264818; doi:10.1186/s12890-024-03172-w)
Supplement: Supplementary file 2 — Supplementary Material 2 [file 12890_2024_3172_MOESM2_ESM.docx]

**Table** **S2** Hosmer-Lemeshow test of models for predicting AE-IPF utilizing combined risk factors.

| **Models** | **X** | ***P*** |
| --- | --- | --- |
| Model 1 | 5.118 | 0.745 |
| Model 2 | 2.949 | 0.938 |
| Model 3 | 10.615 | 0.225 |
| Model 4 | 2.943 | 0.938 |
| Model 5 | 8.092 | 0.425 |

Model 1: PH + honeycombing + whole lung volume; Model 2: PH + honeycombing + whole lung volume + FVC% pred; Model 3: PH + honeycombing + whole lung volume + DLCO% pred; Model 4: PH + honeycombing + whole lung volume + FVC% pred + DLCO% pred; Model 5: PH + honeycombing + FVC% pred + DLCO% pred
