# Supplementary material for: Identification of risk factors for acute exacerbation of idiopathic pulmonary fibrosis based on baseline high-resolution computed tomography: a prospective observational study
Source: BMC Pulm Med. 2024 Jul 19;24:352. doi: 10.1186/s12890-024-03172-w (PMC11264818; doi:10.1186/s12890-024-03172-w)
Supplement: Supplementary file 3 — Supplementary Material 3 [file 12890_2024_3172_MOESM3_ESM.docx]

**
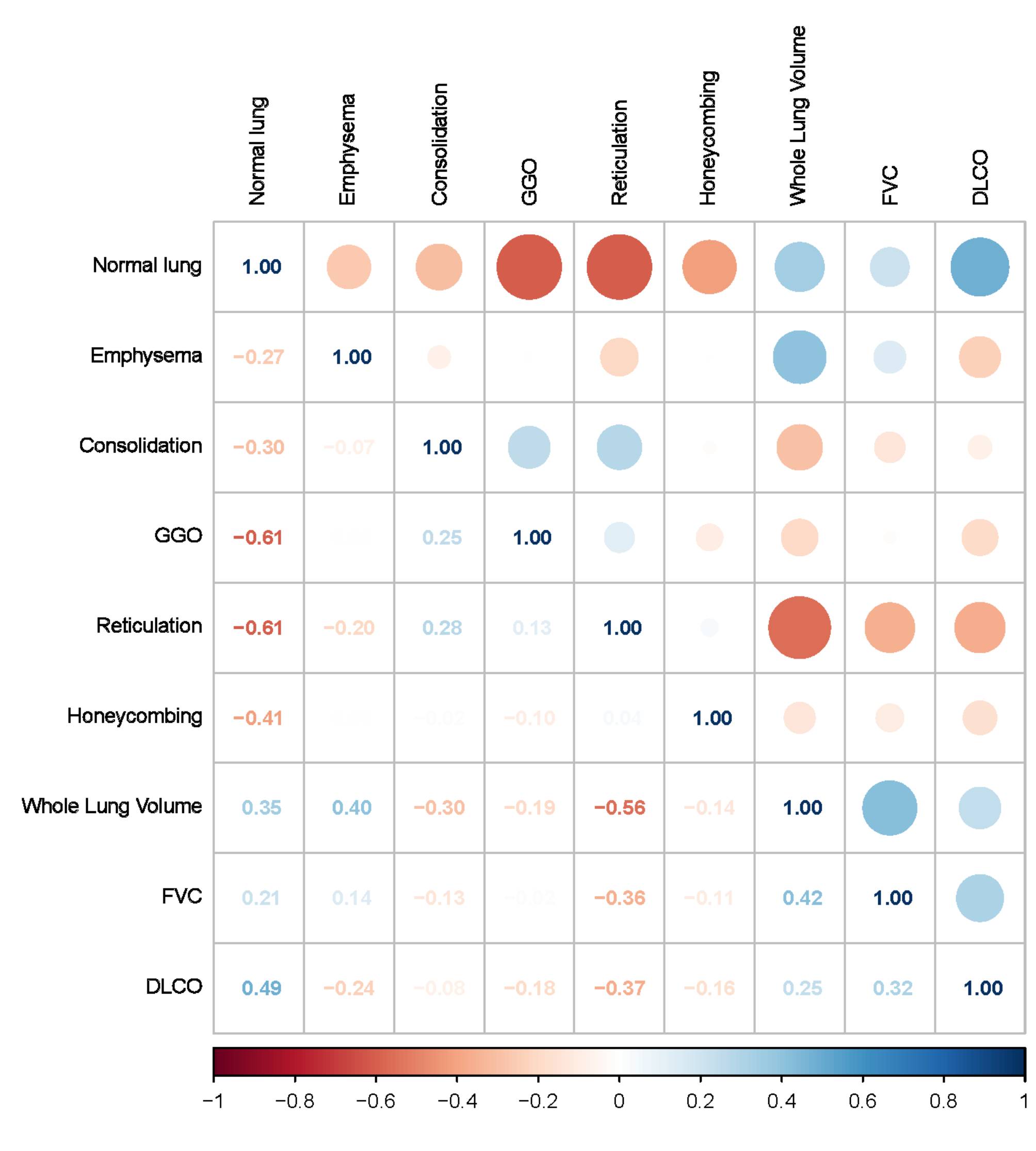
Supplementary figure 1.** Associations between CT-derived quantitative parameters and PFTs. GGO, ground-glass opacities.


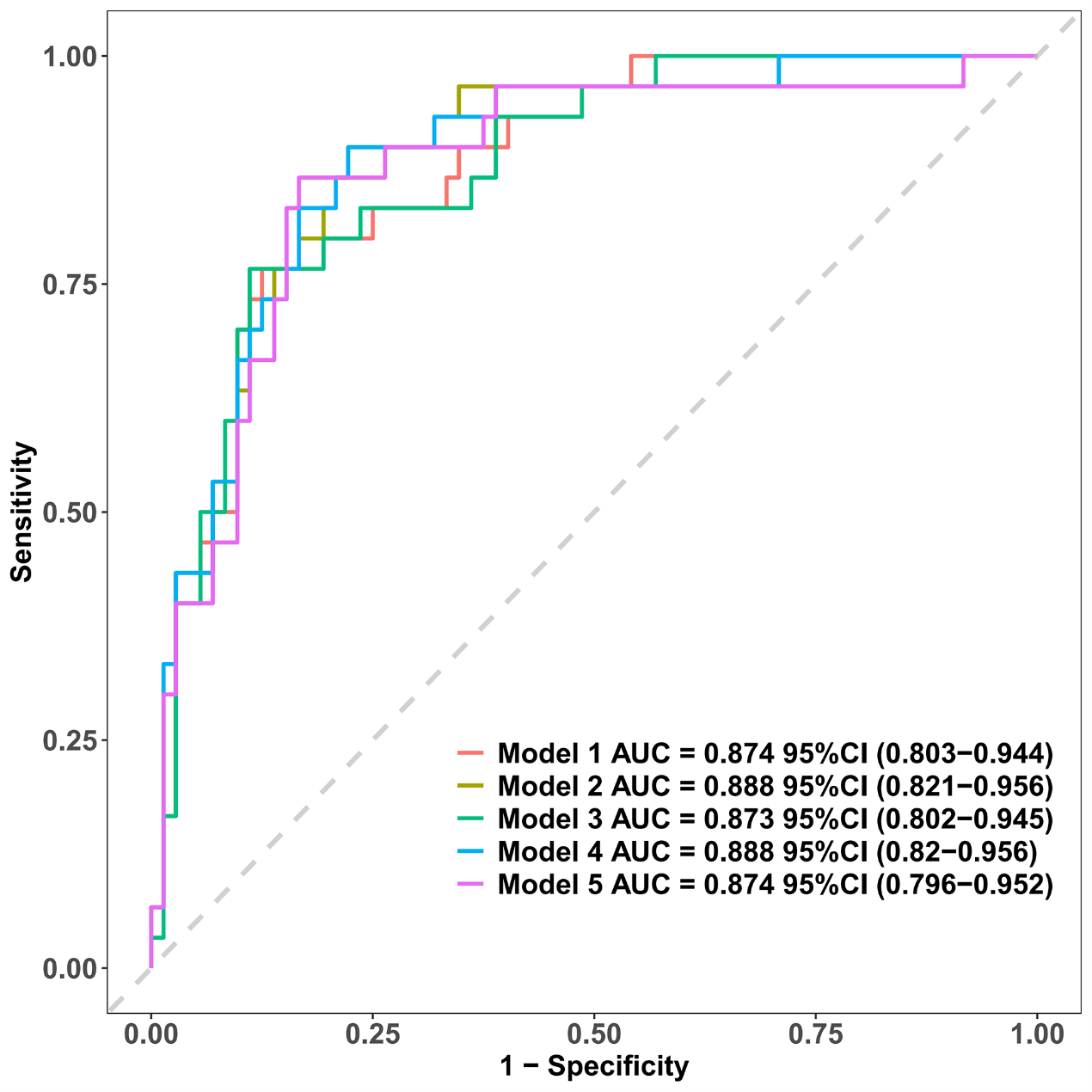


**Supplementary figure 2** The ROC curves of models for predicting AE-IPF.


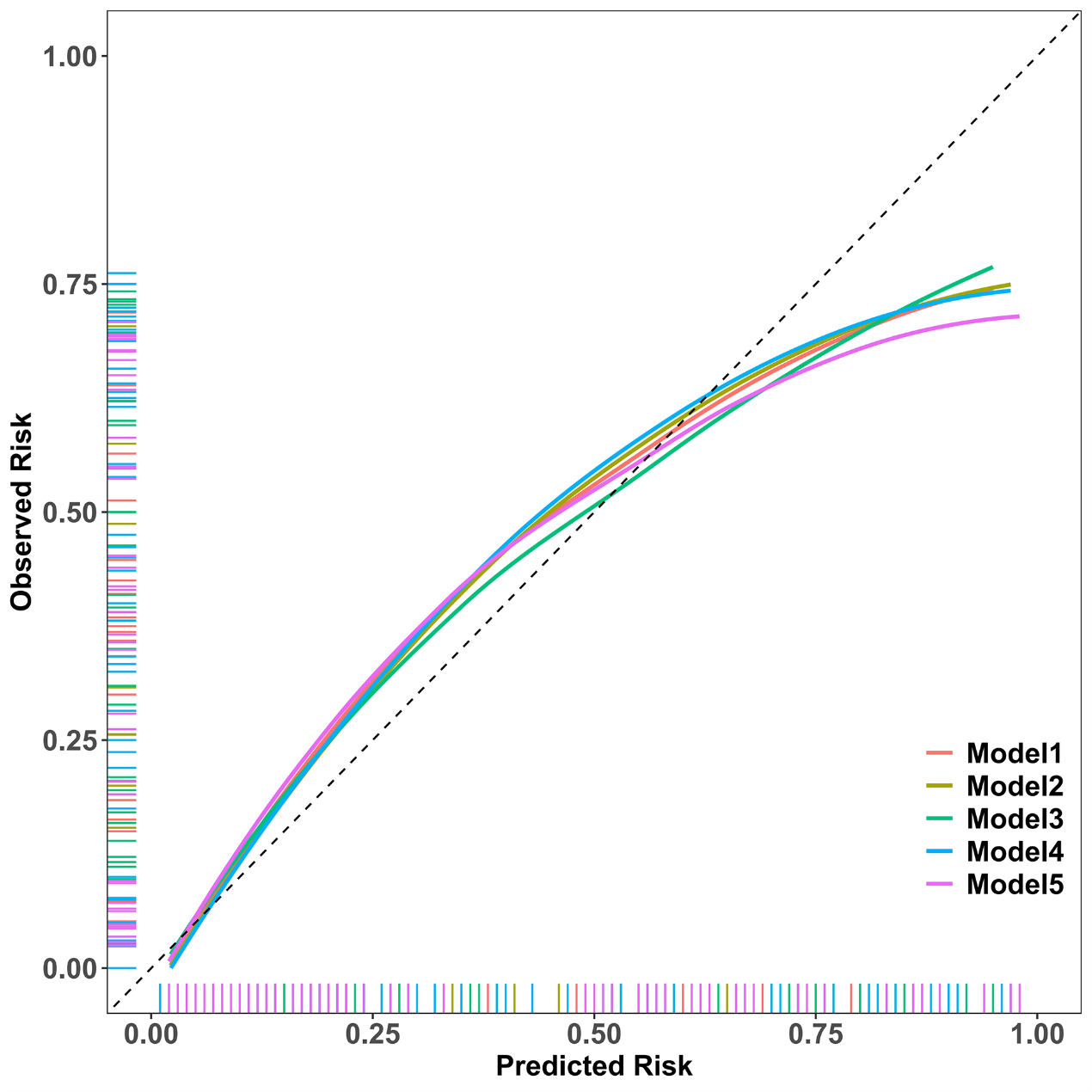


**Supplementary figure 3** The calibration plot of models for predicting AE-IPF.


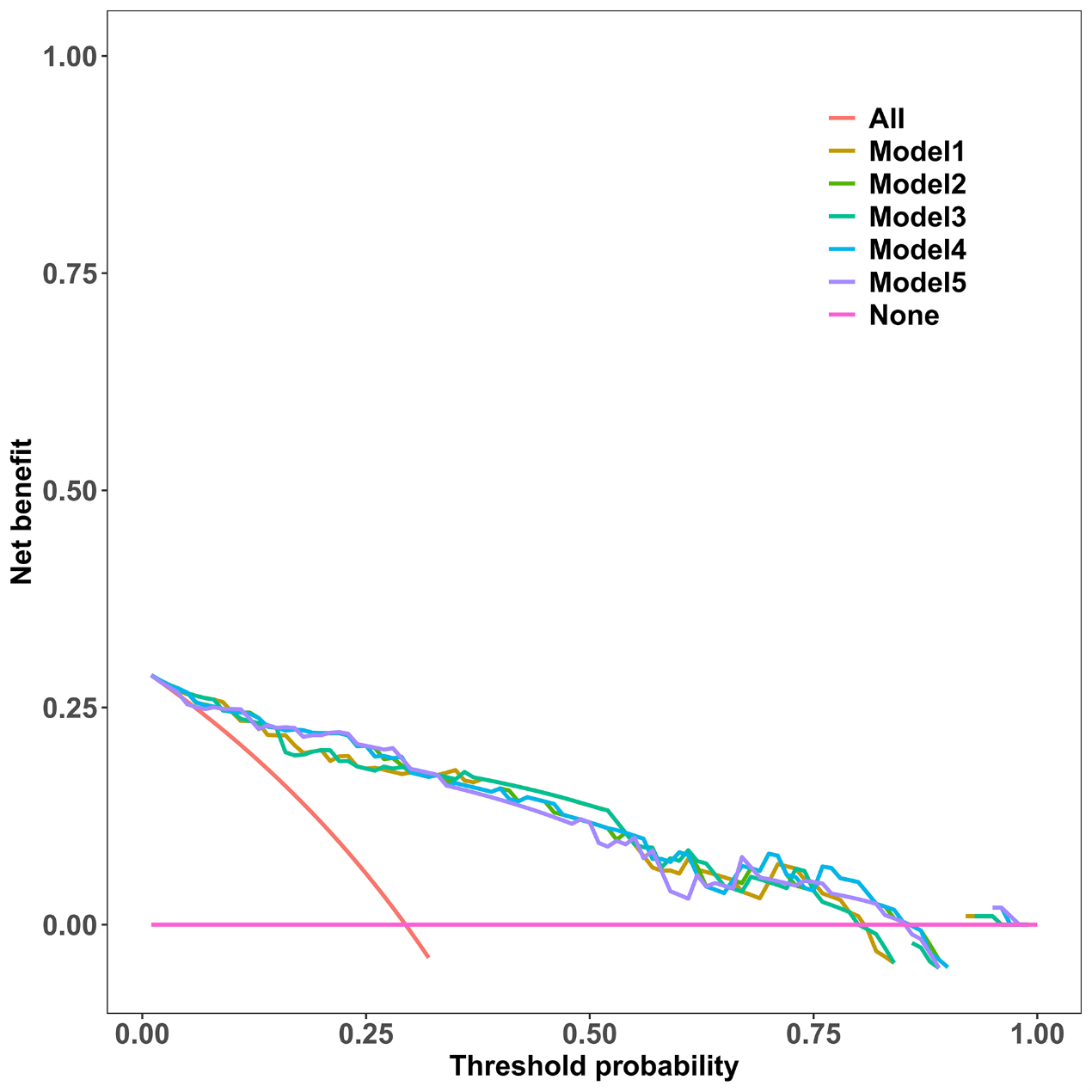


**Supplementary figure 4** The decision curve analysis of models for predicting AE-IPF.


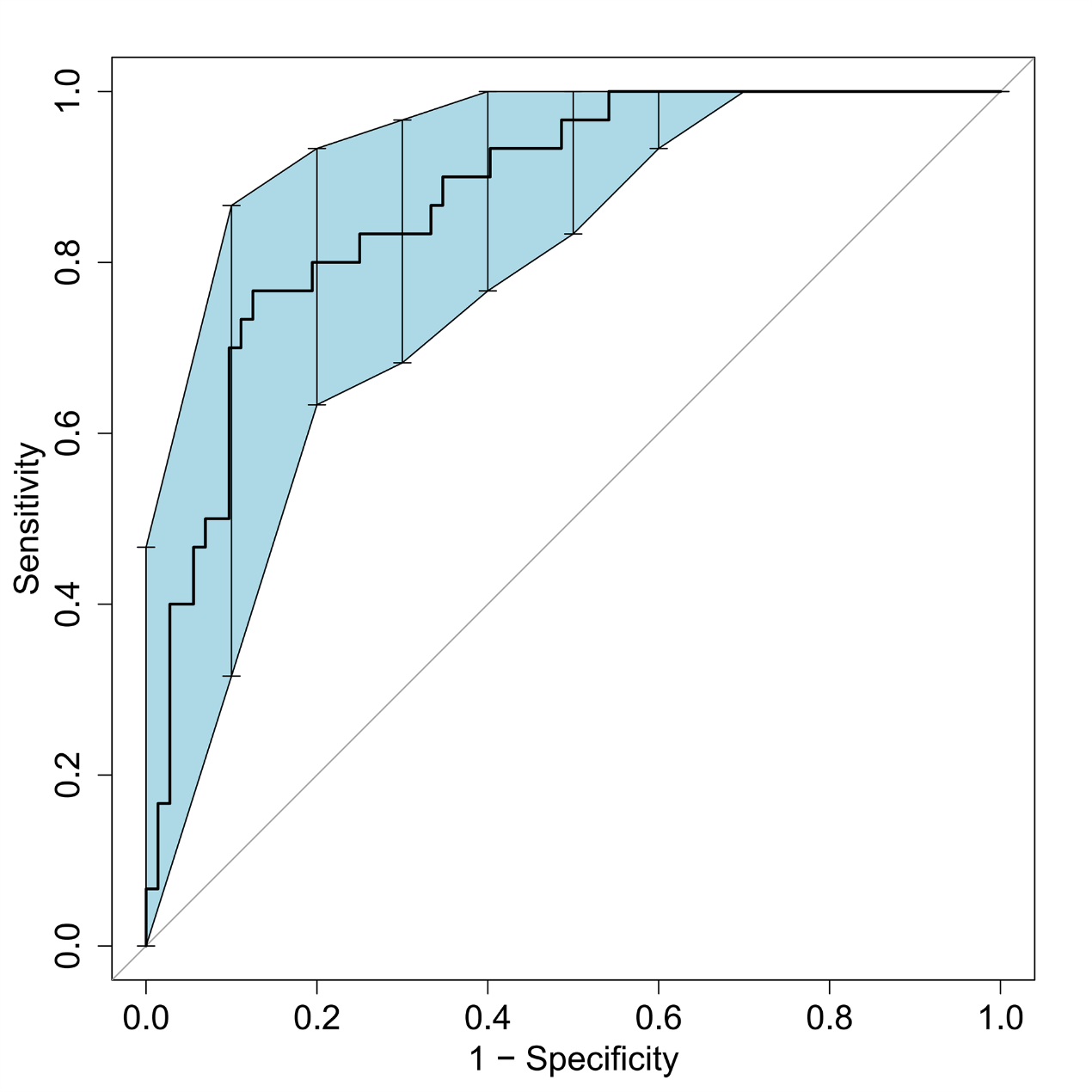


**Supplementary figure 5.** The internal validation using bootstrapping with 500 resamples. Bootstrapped ROC performance: 95% CI:0.792, 0.932.


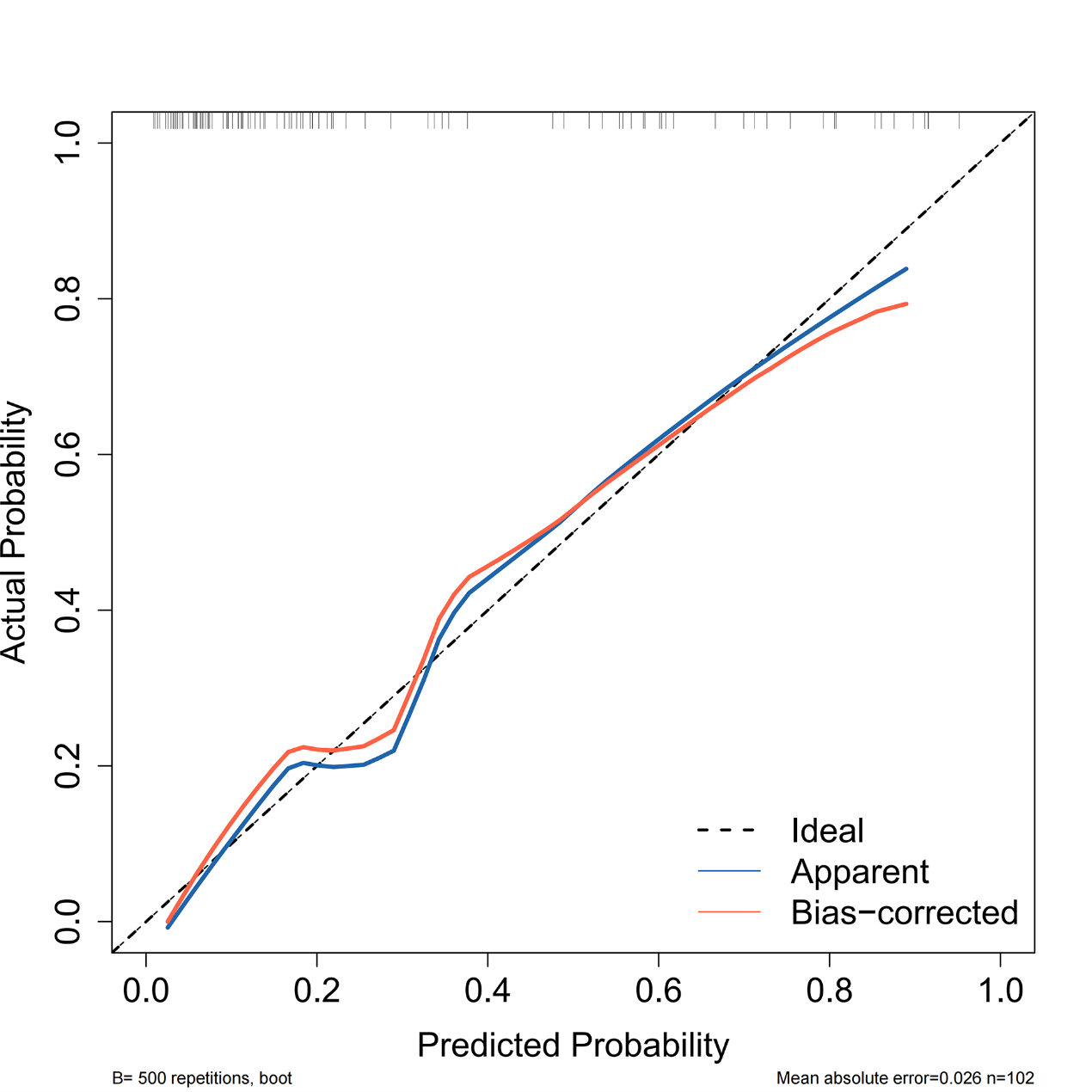


**Supplementary figure 6.** The calibration plot for internal validation of the model.


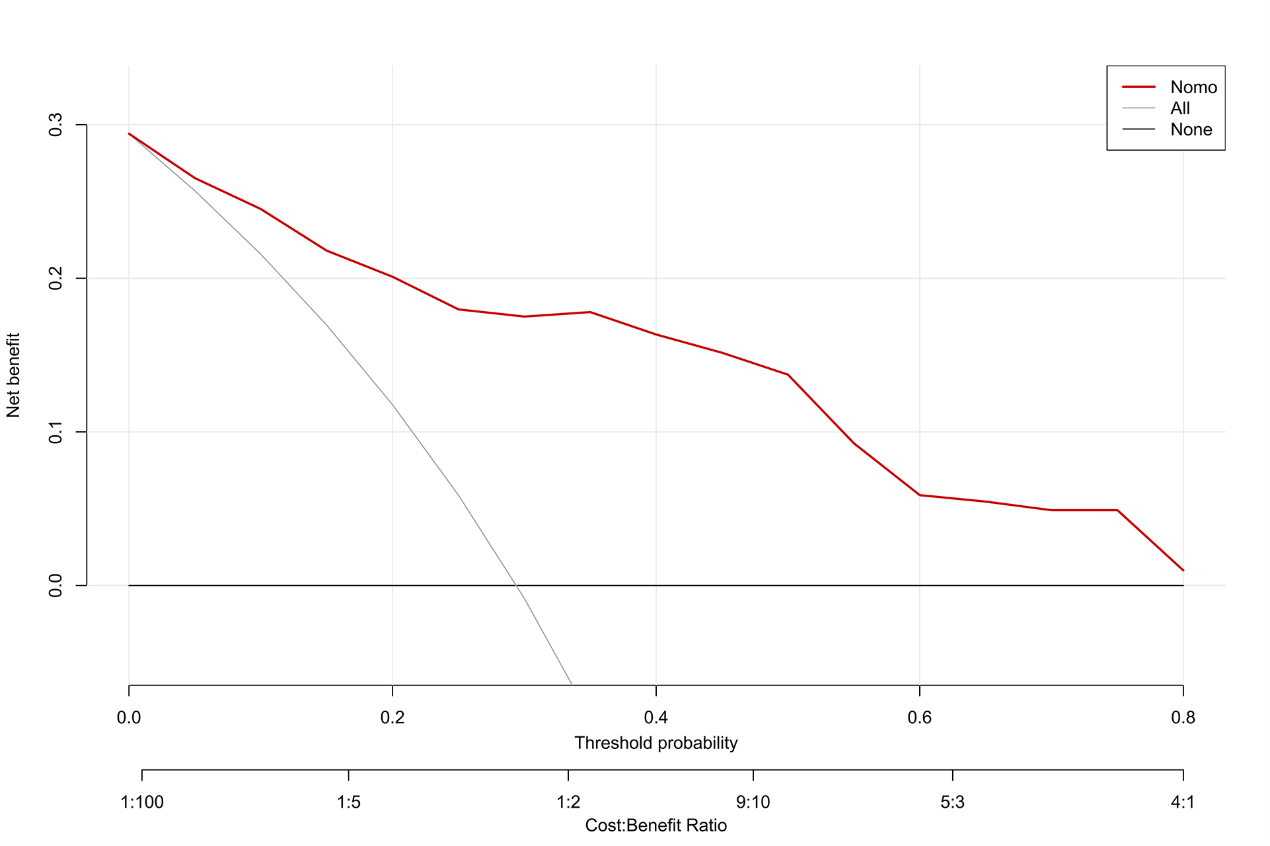


**Supplementary figure 7.** The DCA curve for internal validation of the model.
